# The Impact of Nostalgia Proneness on Online Donation Willingness: The Mediating Effect of Consumer-Brand Relationship

**DOI:** 10.3389/fpsyg.2022.927330

**Published:** 2022-06-13

**Authors:** Yi Zhang, Wenxia Tao

**Affiliations:** School of Economics and Management, Shanghai Institute of Technology, Shanghai, China

**Keywords:** nostalgic feelings, familial utility, emotional utility, trust, relationship commitment, online donation willingness

## Abstract

The rapid outbreak of COVID-19 wreaked havoc and brought a pause to the normal lives, and the labor market and human livelihoods were strongly negatively affected because of it. The emergence of groups that were unable to withstand various pressures has increased the appeal of donation behavior to a certain extent. Therefore, under the impetus of COVID-19 and digital background, online donation represented by Waterdrop financing has become popular. In the common difficult period, how to improve an individual’s willingness to donate online has become an urgent problem to be solved. To address this issue, on the basis of previous literature, we proposed a research hypothesis and a theoretical model of “nostalgia-relationship variables-donation”. After that, we determined the measurement scale, conducted a large sample survey, and finally conducted hypothesis testing through confirmatory factor analysis and structural equation modeling analysis. Through the above analyses, the study reached the following conclusions: the main influence factors of personal nostalgic proneness are insecurity, past experience, loneliness, and recovery from grief, among others. There is a positive causal link between nostalgia proneness and familial utility intensity and emotional utility intensity. The greater the degree of the nostalgia intensity of the donor, the more the trust placed in charitable organizations. The donors’ relationship commitment to charitable organizations significantly influences their online donation willingness. The main source of relationship commitment consists of emotional intensity, followed by trust, and finally, familial intensity.

## Introduction

The rapid outbreak of COVID-19 in 2019 caused serious destructive consequences to the labor market and human livelihoods. In contrast, it accelerated the pace of social digital transformation and enhanced consumers’ sympathy and nostalgia.

Foreign studies have shown that, on the background of COVID-19, donation activities have increased and that donations are mainly aimed at the unemployed and the poor (Bin-Nashwan et al., 2020). In the era of digital consumption, the donation channel of charitable organizations has gradually shifted from traditional online donation to donation network platforms (such as Waterdrop financing), and the status of the consumer–brand relationship is still important. However, nonprofit organizations themselves face some social challenges, and online donation may be more challenging. First, it is difficult for the public to trust charitable organizations. Second, the current national law lacks a perfect supervision system of online donation institutions. Finally, there are still imperfections behind the rapid development of small- and medium-sized charitable organizations. This will weaken the willingness of donors to continue to contribute to charitable organizations. Therefore, during difficult times around the world, how to encourage more people to participate in philanthropy has become a major issue of concern to Chinese and foreign scholars. Scholars at home and abroad have conducted more research on the brand relationship of for-profit organizations, but the exploration of donor–brand relationships of nonprofit organizations in the digital age needs to be deepened.

With the help of the research paradigm of consumer behavior, this article attempts to analyze the nostalgia from “customers” to “donors” from the micro perspective of charitable organizations and tries to prove that the nostalgia of donors can affect the donor’s willingness to donate through the relationship between donors and charitable organizations (especially in the dimensions of trust and relationship commitment). Based on the psychological analysis of charity customers, this study designs nostalgic marketing operation strategies for charity managers to attract more new donors, stimulate existing donors to increase the number and frequency of charitable donations, improve repeated donations and donor loyalty, and improve the overall performance and competitiveness of charity organizations.

## Theoretical Foundations

### Donation and Donor Motivation

Domestic and foreign scholars regard individual donation as an individual public consumption behavior and consider individual donors as consumers of nonprofit organizations. Its consumption behaviors are classified as consumer behavior research fields (Sargeant et al., 2006; Degasperi and Mainardes, 2017). Factors of charitable donations are related to age, gender, occupation, income, religion, and so on (Sargeant et al., 2006; Gao et al., 2022). Sargeant et al. (2006) and Liu et al. (2018) summarize three potential determinants of donation behavior: kin utility (benefit for living or remembered relatives and friends), affective utility (such as warmth and happiness from donation) and dominant utility (meaning direct financial benefit to donors).

### Nostalgic Feeling and Its Marketing Application

Consumer nostalgia is often favored by young people (Holbrook and Schindler, 1991; Shields and Johnson, 2016). As for measuring nostalgic feelings, the scales of nostalgic feeling made by Holbrook (1993); Batcho (1995), and Smeekes et al. (2021) were widely cited, and some Chinese scholars including Lu (2008), Liu et al. (2009), He (2010), and Wen and Qin (2019) also dedicated to the development of nostalgia scales. Nostalgia proneness was mainly related to the living environment, personality, life accident, insecurity, past experience, and loneliness (Ford and Merchant, 2010; Wang et al., 2011; Juhl et al., 2020). Regarding consumer nostalgia in marketing, it was mainly reflected in the decision-making of consumers, brand building, product design, and advertising development (He, 2010; Ju et al., 2016; Fan et al., 2020). The studies by Wang (2010) and Kittikorn and Phanom Klee (2019) showed that stylish nostalgia, technology nostalgia, and nostalgic stories had a regulation effect on consumers’ nostalgia proneness and purchasing desire. The empirical studies by Marchegiani and Phau (2010) and Putra and Fariz (2020) illustrated that personal nostalgia intensity has a positive influence on brand awareness, attitudes, and purchase intention. Loveland et al. (2010) and Hajlaoui and Gharbi (2020) indicated the relationship between brand preference and nostalgic feelings through empirical studies. On the basis of nostalgic proneness, Reisenwitz et al. (2004) and Özhan and Akkaya (2020) depicted that consumers would produce senses of nostalgia by stimulation of nostalgia advertising, which would produce further nostalgic consumption.

### Nostalgic Feeling and Donation Behavior

Sargeant et al. (2006); Bekkers and Wiepking (2007), and Juhl et al. (2020) had shown that the psychological variation of donors as an important factor influencing donor behaviors and nostalgic feelings is one of the common emotional factors. Sargeant (1999) and Sargeant and Woodliffe (2007) thought that individual donations affect both internal and external factors and that donors need to assess their past experiences before they donate. Thus, donors are more willing to donate to charitable organizations that embody their past experiences. Sargeant et al. (2006) and Liu et al. (2018) summed up the internal and external factors for the three categories, friend and relative utility, emotional utility, and dominant utility, and assumed trust and commitment as key variables that decide donation behaviors. On the basis of the studies of Sargeant (1999), Sargeant et al. (2006), Sargeant and Woodliffe (2007), Merchant and Ford (2008) and Ford and Merchant (2010), through an empirical study, demonstrated how nostalgic feelings that are related to past experiences of donors influence friend and relative utility, donation motives, and emotional utilities, thereby ultimately affecting the donation willingness of donors.

The donate model of Sargeant (1999) and Sargeant and Woodliffe (2007) involved evaluations of past experiences of individuals, but it is only a theoretical model. The empirical demonstration of Ford and Merchant (2010) showed nostalgic proneness of donation willingness. However, the model just involved nostalgia proneness but not nostalgia intensity. On the one hand, many other influencing factors of nostalgic proneness need to be explored on the generalization that nostalgia variances in the model are still small. On the other, donation behaviors are simply replaced by online donation willingness in this model and the frequency, quantity, and loyalty of donation among donation behaviors are not involved. Furthermore, trust factors influencing donation proposed by Sargeant et al. (2006) and Degasperi and Mainardes (2017) were not taken into consideration in this model. Kim and Childs (2020) also suggested that willingness to donate may increase when nostalgia for past experiences in charity appeals matches other benefits.

### Future Research Trends

Many existing bodies of literature have studied the relationship between demographic variables and donation behaviors, most of which deeply discussed donation motivation, but the psychological and emotional motivations of donors are rarely involved. Research studies based on marketing perspectives of customer relationships are even less. As for studies on how nostalgic feelings of donation affect the decision-making of donation (consumption), nostalgic feelings are horizontally divided into individual nostalgia and collective nostalgia in the existing literature, whereas discussion from a longitudinal perspective is unusual. Meanwhile, the generation mechanism of nostalgic feelings and influencing factors are often confused and lack hierarchical discussions. Therefore, it is widely accepted that nostalgic feelings are directly related to donation decision-making and that the tiny minority of discussions is related to specific conversion mechanism and intermediary utility in nostalgia.

## Research Hypothesis and Conceptual Model

### Research Hypothesis

#### Antecedents of Nostalgia Proneness

Antecedents of nostalgic feelings involve two aspects, indirect factors and direct trigger factors. The indirect factor refers to factors that affect individual nostalgia proneness, including both external factors, such as society and culture, and personal factors such as individual experience, personality, age, and so on; the direct trigger factor refers to the nostalgic stimulation developed by charitable organizations.

Sense of insecurity is related to nostalgia proneness to some extent. It means that consumers, driven by insecurity, would like to choose the products or brands they trust and know well and form affection or attachment to them, thereby providing an incentive for nostalgic consumers. During a period of crisis or social unrest, nostalgia allows people to seek comfort, gives people a comfortable and friendly emotion, and even becomes the hearted sanctuary (Liu and Zhou, 2009; Martin, 2021). Nostalgia can generate a lot of positive emotions, such as warmth, joy, gratitude, friendship, satisfaction, and sweetness, that could repair negative emotions, filter out unpleasant factors, and strengthen the self-identity of an individual. These positive emotions will comfort those who are uneasy and disgusted with the status quo. For one thing, the more the individuals feel senseless, the more they feel insecure. Then, individuals are more likely to expect to return to the past and live in meaningful conditions with nostalgia. For another, without an identity in the society, individuals tend to achieve self-esteem and self-identity and fulfill self-affirmation *via* nostalgia, thereby helping to reduce the sense of insecurity.

Therefore, we postulate hypothesis 1 (H1): Sense of insecurity in real life has a significant positive impact on personal nostalgia proneness.

Nostalgia is affected by past experiences. The more intense the past experience, the deeper the memory of it (Baumgartner et al., 1992; Holbrook, 1993; Del Líbano et al., 2021). In addition, the more important the things and objects, the more profound the memories stay. Consequently, individuals would be more likely to recall the past, and then more sense of nostalgia for these events is recalled.

Thus, we postulate H2: Past experiences related to charitable organizations have a significant positive impact on personal nostalgia proneness.

Nostalgia is affected by loneliness. In the past, people spent more time with their families and were closely related to social organizations. However, because of the rapid pace of life at present, people are prone to feeling less sense of belonging and sense of security than ever before. When people could not achieve their desire that is expected to enhance their relationship with family and social organizations in real life, people would feel frustrated, depressed, meaningless, and lonely. Naturally, nostalgia, as the sanctuary, could help individuals recall their past memory with family and friends and rebuild memories of happiness when people feel lonely.

In conclusion, we postulate H3: Loneliness has a significant positive impact on personal nostalgia proneness.

Recovery from the sadness of the death of friends and relatives is related to nostalgia. On the one hand, people become quite nostalgic because of past memories and things when their lost loved ones cross their mind. On the other hand, people who felt sad when nostalgic would tend to donate to charity in honor of relatives and in order to recover from their grief.

To this end, we postulate H4: Recovery from grief has a significant positive impact on personal nostalgia proneness.

#### Nostalgia Proneness and Nostalgia Intensity

Nostalgia proneness refers to individual views and attitudes toward the past, and is implicit and stable; nostalgia intensity represents the degree of nostalgia toward nostalgic objects (such as the past and remembrance of things of past times) under external stimulation and is explicit, mutable, and transient.

The degree of nostalgia intensity in the donation scene is shown by emotional utility and friend and relative utility in donation motivation: nostalgic emotional intensity is performed by the size of emotional utility in donation motivation of donors; the intensity of memorizing relatives and friends is reflected through the size of relatives and familial utility.

Influenced by environmental changes brought about by COVID-19 and annual growth, people would increasingly expect to return to the past (Baker and Kennedy, 1994) to cherish the memory of past experiences and people (Goulding, 2001) than ever before (Del Líbano et al., 2021). Once people think of the past, they are often accompanied by feelings of sweetness and sourness: on the one hand, they feel happy when they recall a sweet memory; on the other hand, they would be sad because they could not go back the past by contrasting the reality. However, such feelings tend to be sweet, warm, and comfortable. With charitable donations, people would feel quite a close relationship between themselves and the people they miss when someone thinks of their lover. This kind of intimate relationship would lead to their donations (Sargeant et al., 2006). Under this circumstance, they are likely to obtain a connection with the past lovers when donating in their own names (Fischer et al., 1996; Stuhler, 2017; Myrick and Willoughby, 2019). Therefore, donations act as a platform for an individual to experience nostalgia and, thereby, to bridge the distance with their loved ones. For instance, someone’s relative died of cancer, so he donated to a cancer charity on behalf of the dead, which would make him feel more intimate with the deceased (relatives or friends). This is the relative utility described by Sargeant et al. (2006).

Therefore, we hypothesize H5: Individual nostalgia proneness has a significant positive impact on relative and friend intensity.

Individual nostalgic experience is reflected not only by the memory of people and goods but also in the remembrance of the past. For example, universities stimulate the memory of school graduates through various ways to appeal to their senses of nostalgia, consequently donating to their universities (Appleton-Knapp et al., 2004; Del Líbano et al., 2021). In terms of students, their donations to their old schools would enable them to rebuild yesterday and produce a nostalgic experience that would bring about pleasantness, happiness, and a sense of wellbeing to them (Holak and Havlena, 1998). Overall, individual nostalgia will produce emotional utility, which is the very emotional utility described by Sargeant et al. (2006).

Overall, we hypothesize H6: Individual nostalgia proneness has a significant positive impact on emotional intensity.

#### Nostalgia Intensity and Brand Relationships

##### Nostalgia Intensity and Brand Trust

Trust is one of the important factors that decide the level of donation (Sargeant et al., 2006; Bilgin and Kethüda, 2022). The level of individual nostalgia has a certain impact on trust as well. Lu (2008), in her study on the relationship between individual nostalgia and trust of Chinese time-honored brands, indicated that there is a certain positive correlation between trust level and individual nostalgia according to her own nostalgia scale. Hidayati (2021) also mentioned it. Li (2007), in an article about the restoration of Chinese time-honored brands, concluded that nostalgia has a negative correlation with trust using the nostalgia scale made by Holbrook (1993). He (2010), using his CHINOS scale, measured the relationship between nostalgia proneness and brand trust and showed that domestic brands have a good predictive validity to nostalgic proneness through empirical research. Wen et al. (2019) conducted a study on nostalgic restaurants. The results show that nostalgia has a significant positive impact on brand trust. To this end, we hypothesize the following:H7: Relative and friend intensity of individual donors has a significant positive impact on their trust level in charitable organizations. H8: Emotional intensity of individual donors has a significant positive impact on their trust levels for charitable organizations.

##### Nostalgia Intensity and Relationship Commitment

Commitment is a continued desire to maintain a valuable relationship (Morgan and Hunt, 1994; Rather and Sharma, 2017). The depth of the relationship between donors and charities would affect the level of their commitment to charities (Sargeant and Lee, 2004; Akrout and Nagy, 2018). That is to say, the higher the emotional intensity and relative memorization, the higher the commitment levels of donors to charitable organizations. Individuals produce emotional intensity and relative intensity through nostalgic experiences, thereby enhancing commitment levels of origination (Sargeant et al., 2006). In this sense, donation has become a platform where donors could relive the past and become closer to their departed relatives. To this end, we hypothesize the following: H9: Friend and relative intensity has a significant positive impact on the commitment of individual donors to charitable organizations, and that: H10: Emotional intensity has a significant positive impact on the commitment of individual donors to charitable organizations.

#### Brand Trust and Relationship Commitment

Chaudhuri and Holbrook (2001) and Hidayanti and Nuryakin (2018) found that there is a positive relationship between brand trust and brand promise. It means that brand trust is the core pilot of the brand promise, which could be shown in many studies. It is trust that drives the form of commitment. In general, commitment would always involve self-sacrifice to some extent, and it is unlikely to occur without trust (Morgan and Hunt, 1994). Wong and Sohal (2002) made a further explanation of the relationship between trust and commitment; thus, it can be seen that trust is a of prerequisite for commitment. Sargeant et al. (2006) also pointed out that commitment is a function of trust. Additionally, Sargeant and Lee (2004) and Sargeant et al. (2006), in two articles on influencing factors of donation, regarded trust as the important factor determining the donation level and showed that the trust level of donors to charities would affect the level of commitment, thereby affecting donation behaviors. Ko et al. (2014) pointed out by empirical research that trust and commitment have a significant positive impact on donation behavior.

In China, the crisis of confidence is one of the major obstacles to the development of charitable donations. With the development of charities in China, trust problem has continuously emerged in the charitable field. Netease, one of most famous Internet companies, ended up with the Netease online donation platform of China Red Cross Society on 14 May 2008, while Netease began to cooperate with the Liao Bingxiong Humane Fund Management Committee. In addition, an increasing number of netizens give rise to questions about the amount and channels of donation of some celebrities such as Yu Qiuyu and Chen Guangbiao. What is worse, the development of charity career, which is particular in the development of individual charity level, has undergone its bottom because of Guo MeimeiEvent in 2011 and ten thousand-yuan catering invoice events in Shanghai Luwan District Red Cross. Based on the above, trust is a necessary condition for the public to donate to charitable organizations in China, since trust would affect the degree of relationship commitment between individual donors and charitable organizations, thereby affecting online donation willingness and donation level.

Therefore, we postulate H11: The degree of trust of individual donations on nonprofit organizations has a significantly positive correlation with commitment level.

#### Relationship Commitment and Online Donation Willingness

The impact of commitment on purchase intention and brand loyalty had already been found in the marketing research field previously. Morgan and Hunt (1994), Bansal et al. (2004), and other scholars believed that it is customer commitment that is the core concept to maintaining and developing brand relationships, since it is the key psychological driving force that contacts customers and sales organizations. Many existing empirical studies have proved that there is a positive correlation between customer commitment and purchase intention and brand loyalty (e.g., Anderson and Weitz, 1992; Morgan and Hunt, 1994; Garbarino and Johnson, 1999; Wang et al., 2019; Khodabandeh and Lindh, 2021). In terms of donors, donors, according to their own past experiences and other factors, measured whether the public welfare of nonprofit organizations enables donors to generate a sense of trust and develop further relationship commitment, accordingly deciding whether or not to donate to charitable organization or continue to donate (Sargeant et al., 2006).

To this end, we hypothesize H12: The commitment level of donors to charitable organizations has a significant positive correlation with online donation willingness.

### Conceptual Model

**FIGURE 1:**
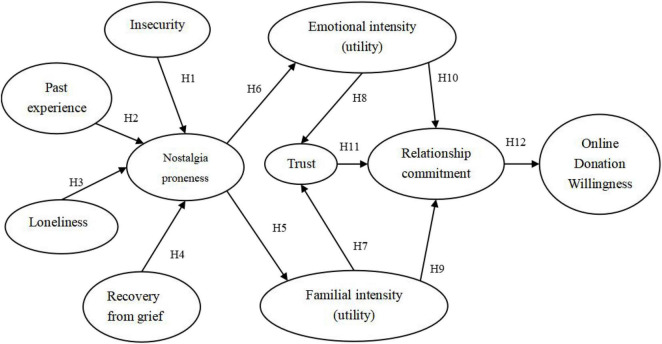
Online donation intention model.

The donation model of Sargeant et al. (2006) is an important reference for this study. As for the discussion about emotional influencing factors, past experience, loneliness, and recovery from grief involved in the nostalgia proneness of influencing factors by the donation model of Merchant and Ford (2008) and Ford and Merchant (2010), are among those mentioned in this study for reference. Based on the abovementioned factors, the theoretical model is proposed and consists of four parts (shown in Figure 1). First, nostalgia proneness influencing factors are composed of insecurity, past experience, loneliness, and grief recovery. Second, nostalgia proneness and nostalgia intensity (emotion intensity and relative and friend intensity) are considered as two levels of nostalgic feelings, and nostalgic proneness would be performed as nostalgia intensity when charitable organizations’ irritants are evoked. Third, nostalgia intensity is characterized by donation motivation of donors, that is, the degree of donation motivation (emotional utility intensity and relative and familial utility intensity) affected by nostalgic irritants of charitable organizations represents nostalgia intensity. Fourth, nostalgia intensity affects donation behaviors (represent donate willingness) through an intermediary played by the quality of donor-charitable organization relationship (trust and relationship commitment).

## Methods and Findings

### Construct Scales

The measurement scale is the basis of the empirical research because the credibility and validity of the measurement scale would directly affect the meaning and value of the entire study. The main part of the test item in this article includes ten parts. The online donation willingness adopts the scale designed by Sargeant et al. (2006); Hou (2009), and Ye et al. (2015), with a total of three items. The measurement of commitment relationship adopts the scale modified and used in the article by Sargeant et al. (2006) and Hou (2009), with a total of five items. Trust refers to and modifies the scale proposed by Sargeant et al. (2006); Hou (2009), and Bauer and Freitag (2018), with a total of four items. The emotional intensity adopts the scale designed by Sargeant et al. (2006) and Goto and Schaefer (2020), with three items in total. The scale modified and used by Sargeant et al. (2006) was used to measure the intensity of relations and friends, with three items in total. Nostalgia proneness adopts the scale designed by Batcho (1995), Lu (2008); He (2010), Merchant and Ford (2008), Ford and Merchant (2010), and Cheung et al. (2017), with 12 items in total. The measurement of insecurity adopts the scale modified and used in the article by Collins and Read (1990) and Wang (2010), with a total of four items. Loneliness refers to and modifies the scale proposed by Russell et al. (1980) and Auné et al. (2019), with a total of nine items. Recovery from grief refers to and modifies the scale proposed by Carnelley et al. (2006), Merchant and Ford (2008), Ford and Merchant (2010), and Nolan and Hallam (2019), with a total of three items. Past experience refers to the scale proposed by Baumgartner et al. (1992); Schulkind et al. (1999), Merchant and Ford (2008), Ford and Merchant (2010), and Afaq et al. (2020), with two items in total.

### Date Collection

A large sample survey was conducted from February to April 2022. Since we studied the donation behavior of respondents, we considered the donors of online donation platforms as the survey object. To this end, we used the following methods to conduct a questionnaire survey: first, 750 individual donors were randomly selected to conduct a questionnaire survey through the donor information from nonprofit organizations such as Waterdrop financing, easy financing, and worry-free financing; therefore, 440 questionnaires were withdrawn within the specified time limit; after eliminating invalid questionnaires, there were 394 valid questionnaires, and the effective rate was 52%.

The analysis of large sample survey included descriptive statistics, variable data description, CITC, reliability, one dimensionality, and validity. Project descriptive statistics is mainly conducted for preliminary testing of variable projects. CITC (total correlation of correction items) and Cronbach’s α reliability coefficient are used for the reliability coefficient of purification measurement items and test measurement items, respectively. The factor analysis, at the structural level, was conducted to verify the single-dimensional variables. In addition, we tested the fitting of large sample data and theoretical hypothesis model with the AMOS26.0.

### Descriptive Statistical Analysis

First, the descriptive statistics were conducted to measure the measured values of variables and analysis results, which include the mean of each variable, standard deviation, and so on. As it can be seen from the Table 1, the average of each variable could basically reflect the degree of concentration since it should tend to the middle values and not have extreme averages. From variance evolution, each variable has a relatively large variation quantity, and the senses in which interviewees complete the questionnaires have some differences, so the identification degree of subject is relatively high.

**TABLE 1:** Descriptive statistics of variables.

Variable	Item	Minimum	Maximum	Average	Standard deviation
Online donation willingness	ID1, ID2, and ID3	1.00	7.00	4.64	1.02
Relationship commitment	C1, C2, C3, and C4	1.00	7.00	4.09	1.23
Trust	T1, T2, and T3	1.00	7.00	4.43	1.11
Emotional intensity	EI1, EI2, and EI3	1.00	7.00	4.73	1.31
Relatives and friends intensity	FI1, FI2, and FI3	1.00	7.00	4.04	0.99
Nostalgia proneness	PN1, PN2, PN3, PN4, PN5, PN6, PN7, PN9, PN10, PN11, and PN12	1.00	7.00	4.13	1.04
Insecurity	IS1, IS4, and IS4	1.00	7.00	4.59	1.05
Loneliness	L1, L2, L3, L4, L6, L7, L8, and L9	1.00	7.00	4.76	1.03
Recovery from grief	R1 and R2	1.00	7.00	3.99	1.28
Past experience	PE1 and PE2	1.00	7.00	4.28	0.98

### Reliability and Validity Analysis of Large Sample Survey Data

By CITC, reliability, and validity analysis of the large sample, we found that C3 and C5 in relationship commitment, T4 in trust, PN8 and PN11 in nostalgia proneness, L5 and L6 in loneliness, R3 in recovery from grief, and IS2 in insecurity should be deleted. From the item analysis of both the large sample and the small sample, the measurement items of all variables were optimized, after which 39 optimized measurement items in total are shown in the table below. Then, a statistical analysis was conducted according to the optimized measurement items in the following text.

### Confirmatory Factor Analysis

The factor standardized loading, Cronbach’s α, and fitting index of measurement model are shown in Table 2. The measurement model and data are satisfactory because they passed the AMOS test and the adaptation standard or critical value.

**TABLE 2:** Confirmatory factor analysis of constructs.

Construct	Item code	Item	Standardized loading	*P*-value
Online donation willingness α = 0.903	ID1	(1) I would continuously donate to this charity organization	0.907	0.000
	ID2	(2) I would like to donate to this charity organization	0.885	0.000
	ID3	(3) I would donate to this charity organization again next time	0.915	0.000
Relationship commitment α = 0.887	C1	(1) I think that I have sense of belongingness to this charity organization	0.799	0.000
	C2	(2) I think I am a loyal supporters of this charity organization	0.875	0.000
	C4	(4) I would recommend others donors to donate to this charity organization	0.895	0.000
Trust α = 0.893	T1	(1) I believe that this charity organization could effectively use contribution money (or object)	0.765	0.000
	T2	(2) I believe that this organization is a righteous charity organization	0.845	0.000
	T3	(3) I do not think this charity organization would cheat us	0.874	0.000
Emotion intensity α = 0.738	EI1	(1) I would feel upset without donation, when this charity organization raise donation	0.809	0.000
	EI2	(2) I would be comfort after donating	0.811	0.000
	EI3	(3) I would feel wellbeing after donating to this charity organization	0.712	0.000
Relatives and Friends intensity α = 0.715	FI1	(1) I donate to this charity organization in honor of somebody	0.821	0.000
	FI2	(2) I think the person I know (including the donation targets in my imagination) would benefit from my donation	0.734	0.000
	FI3	(3) I think that my relatives and friends (including the donation targets in my imagination) have certain relation with this organization	0.835	0.000
Nostalgia proneness α = 0.901	PN1	(1) I miss old songs	0.853	0.000
	PN2	(2) I often recall the past experience	0.743	0.000
	PN3	(3) I still like watching the movies and TV plays I used to love	0.863	0.000
	PN4	(4) I could not forget the flavor of food I tasted in my childhood	0.777	0.000
	PN5	(5) I miss the place I used to live	0.784	0.000
	PN6	(6) I miss used schools	0.834	0.000
	PN7	(7) I miss carefree old time	0.763	0.000
	PN9	(9) I miss the old time together with relatives and friends	0.743	0.000
	PN10	(10) I often think of concern and love of relatives and friends in my childhood	0.823	0.000
	PN12	(12) I miss the lifestyle of past people	0.832	0.000
Insecurity α = 0.726	IS1	(1) I do not think my life is stable	0.786	0.000
	IS3	(3) I usually worried that I would be abandoned	0.723	0.000
	IS4	(4) I am lack of confidence in my ability	0.737	0.000
Loneliness α = 0.838	L1	(1) I have few companion	0.838	0.000
	L2	(2) there is no one I could pour out	0.769	0.000
	L3	(3) I feel that I am part of some circle	0.835	0.000
	L4	(4) I am not close to others	0.787	0.000
	L7	(7) No one really understand me	0.843	0.000
	L8	(8) I feel unhappy due to loneliness	0.867	0.000
	L9	(9) The people by my side do not support me	0.842	0.000
Recovery from grief α = 0.823	R1	(1) I often think of departed relatives and friends who used to bring happiness to me	0.712	0.000
	R2	(2) I feel warm when I think of my departed relatives and friends	0.756	0.000
Past experience α = 0.819	PE1	(1) This charity organization clearly recalls my memory	0.820	0.000
	PE2	(2) This organization trigger my memory and make my emotion ups and downs	0.735	0.000
Goodness of fit of the model: *x*^2^ (690) = 1,469.7, *x*^2^/*df* = 2.13, CFI = 0.92, IFI = 0.91, TLI = 0.92, GFI = 0.86, and RMSEA = 0.046				

### Correlation Analysis

Before the structural equation modeling test, a pairwise correlation analysis is conducted to each latent variable first, which is the preliminary test of the hypothesis. In general, the two variables in the assumptions should have a high correlation. Meanwhile, correlation coefficient is statistically significant. The correlation between each latent variable is analyzed with SPSS26.0, and the correlation coefficient between each latent variable is shown in Table 3. It can be seen from the table that each latent variable is significantly correlated; it is basically a significant positive correlation as the assumption we presented above. Therefore, the original assumption is basically verified.

**TABLE 3:** Correlations among latent constructs.

Variable	1	2	3	4	5	6	7	8	9	10
Online donation willingness	1									
Relationship commitment	0.797**	1								
Trust	0.632**	0.638**	1							
Emotion intensity	0.521**	0.563**	0.583**	1						
Relatives and friends intensity	0.327**	0.421**	0.457**	0.307**	1					
Nostalgia proneness	0.019	0.057	0.112*	0.193**	0.187**	1				
Loneliness	−0.168**	−0.174**	−0.147**	–0.086	0.051	0.197**	1			
Past experience	0.142**	0.151**	0.163**	0.193**	0.287**	0.184**	0.081	1		
Insecurity	0.146**	0.169**	0.167**	0.183**	0.187**	0.207**	0.204**	0.173**	1	
Recovery from grief	0.168**	0.142**	0.137**	0.194**	0.141**	0.201**	−0.169*	0.144**	0.189**	1

**Correlation is significant at the 0.01 level. *Correlation is significant at the 0.05 level.

### Hypothesis Test

The theoretical assumptions above are preliminarily demonstrated by a correlation analysis testing. In order to verify the causal relationship between self-variables and dependent variables, AMOS26.0 is used to study the hypothesis testing of the model.

A summary of the hypothesis test is provided in Table 4, and all the assumptions are tested with the path coefficient.

**TABLE 4:** Structural model results and hypothesis tests.

Path in the model	Standardized estimate	standard error	T-values	Support or not
Past experience–> Nostalgia proneness	0.167	0.049	3.422*	support
Recovery from grief–> Nostalgia proneness	0.199	0.053	3.739**	support
Loneliness –> Nostalgia proneness	0.158	0.048	3.310*	Support
Insecurity –> Nostalgia proneness	0.258	0.043	5.967**	Support
Nostalgia proneness – > Relatives and friends intensity	0.227	0.059	3.827**	Support
Nostalgia proneness –> Relatives and friends intensity	0.189	0.052	3.609**	Support
Relatives and friends intensity –> Trust	0.194	0.052	3.74**	Support
Emotional intensity –> Trust	0.247	0.054	4.584**	Support
Relatives and friends intensity –> Relationship commitment	0.302	0.060	5.007**	Support
Emotional intensity–> Relationship commitment	0.591	0.058	10.228**	Support
Trust–> Relationship commitment	0.434	0.059	7.247**	Support
Relationship commitment –> online donation willingness	0.894	0.061	14.709**	Support

**Significant at P < 0.001, *Significant at P < 0.01.

H1: Realistic insecurity has a significant positive impact on personal nostalgia proneness. The path coefficient is 0.258 and the T value is equal to 5.967, which are significant at a confidence level of 0.001. Therefore, the hypothesis is supported. According to this hypothesis, when individuals feel insecurity in real life, they would be likely to be nostalgic and seek comfort. Wang (2010) and Areni et al. (2022) studied the relationship between the two variables. Their research results have been further demonstrated in this study.

H2: Past experiences related to charitable organizations have a significant positive impact on personal nostalgia proneness. The path coefficient is 0.167 and the T value is equal to 3.4227, which are significant at a confidence level of 0.001. Therefore, the hypothesis is supported. According to this hypothesis, the more important the past experience of donors is, the deeply they remember. Memories of past experiences in real life have a more remarkable impact. Belk (1991) and Del Líbano et al. (2021) studied the relationship between the two variables, and further research is conducted in this study to demonstrate their research results. From practices of management, if managers of charitable organizations want to stimulate the nostalgic feelings of donors, they need to ensure that donors have past experiences related to their organizations or their organization appeal.

H3: Loneliness has a significant positive impact on personal nostalgia proneness. The path coefficient is 0.158 and the T value is equal to 3.31, which are significant at a confidence level of 0.001. Therefore, the hypothesis is supported. It means that individuals would become nostalgic and always remember past good times when they feel lonely in real life. Merchant and Ford (2008), Ford and Merchant (2010), and Juhl et al. (2020) studied the relationship between the two variables, and their research results have been further demonstrated in this study.

H4: Recovery from grief has a significant positive impact on personal nostalgia proneness. The path coefficient is 0.199 and the T value is equal to 3.739, which are significant at a confidence level of 0.001. Therefore, the hypothesis is supported. Accordingly, individuals would think of old times spent with their relatives and friends when they recalled them. Nostalgia is considered an important means to overcome grief. The relationship above was rarely mentioned in the previous literature, except Merchant and Ford (2008) and Ford and Merchant (2010). Their research results have been further demonstrated in this study.

H5: Personal nostalgia proneness has a significant positive impact on relative and friend intensity. The path coefficient is 0.227 and the T value is equal to 3.827, which are significant at a confidence level of 0.001. Therefore, the hypothesis is supported. According to the hypothesis, when someone thinks of relatives and spouse, individuals often feel a very close relationship with the people they miss. This intimate relationship would lead to his or her donation (Sargeant et al., 2006) and gets a connection between their past relatives and friends and themselves (Fischer et al., 1996; Stuhler, 2017; Myrick and Willoughby, 2019).

H6: Personal nostalgia proneness has a significant positive impact on emotional intensity. The path coefficient is 0.189 and the T value is equal to 3.609, which are significant at a confidence level of 0.001. Therefore, the hypothesis is supported. Under the hypothesis, the nostalgic proneness of donors would produce a sense of “yesterday once more,” thereby appreciating pleasantness, happiness, and warmth (Holak and Havlena, 1998). Sargeant et al. (2006), Merchant and Ford (2008), Ford and Merchant (2010), and Batcho (2020) conducted such research. Their research results have been further demonstrated in this study.

H7: Relative and friend intensity of individual donations has a significant positive impact on the level of trust on charitable organizations.

H8: The emotional intensity of individual donors has a significant positive impact on the trust level for charitable organizations. The path coefficient of “relative and friend intensity to trust” is 0.194 and the T value is equal to 3.74, which are significant at a confidence level of 0.001. The path coefficient “emotional intensity to trust” is 0.247 and the T value is equal to 4.584, which are significant at a confidence level of 0.001. Therefore, the two hypotheses are supported. According to the two hypotheses, individuals would have more confidence in charitable organizations when a certain nostalgia intensity is aroused and produced by the organizations. It is unusual to find literature on the relationship between nostalgia intensity and trust at present, but the relationship above provides a way for charitable organizations to improve their trust.

H9: Relative and friend intensity has a significant positive impact on the relationship commitment of individual donors to charitable organizations.

H10: Emotional intensity has a significant positive impact on the relationship commitment of individual donors to charitable organizations. The path coefficient between relative and friend intensity and relationship commitment is 0.302 and the T value is equal to 5.007, which are significant at a confidence level of 0.001. The path coefficient between emotional intensity and relationship commitment is 0.591 and the T value is equal to 10.22, which are significant at a confidence level of 0.001. Therefore, the two hypotheses are supported. Under the two hypotheses, donors would have a greater relationship commitment to charitable organizations when nostalgic intensity is aroused and produced by the organizations. Sargeant et al. (2006), Merchant and Ford (2008), Ford and Merchant (2010), and Stuhler (2017) conducted such research in previous years. Their research results have been further demonstrated in this study.

H11: The trust level of individual donors on nonprofit organizations presents a significant positive correlation on donation commitment level. The path coefficient is 0.434 and the T value is equal to 7.247, which are significant at a confidence level of 0.001. Therefore, the hypothesis is supported. According to the hypothesis, donors would produce a greater relationship commitment to charitable organizations because of their increasing trust to the organizations. This relationship had been studied by Sargeant et al. (2006). However, according to Sargeant et al., trust is mainly derived from the behavior of charitable organizations itself, such as organizational performance, organizational feedback, organizational communication and so on; the results of Sargeant et al. (2006) and Kim and Childs (2020) from the perspective of the psychological feelings of donors such as nostalgic feelings are demonstrated in this study.

H12: The level of commitment of donors to charitable organizations presents a significant positive correlation with online donation willingness. The path coefficient is 0.894 and the T value is equal to 14.709, which are significant at a confidence level of 0.001. Therefore, the assumption is supported. According to this hypothesis, the online donation willingness of donors would improve as donors produce a growing relationship commitment to charitable organizations. Based on the context of donation above, we demonstrated the relationship between the commitment and the purchase intention again, which has been researched by many marketing researchers (such as Anderson and Weitz, 1992; Morgan and Hunt, 1994; Garbarino and Johnson, 1999; Wang et al., 2019; Peng et al., 2021).

## Conclusion and Recommendations

### Summary of Findings

As shown in Table 5, the square of path coefficients to assess the variance explained by degrees of endogenous variables is calculated in the model. Cohen (1992) showed that when the model uses multiple independent variables (exogenous variables), approximately 2% of the variance explained that the degrees are relatively small, approximately 15% is moderate, and over approximately 35% is rather bigger. We can draw the following conclusions shown in Table 5:

**TABLE 5:** Variance explained by endogenous constructs.

Endogenous construct		Variance explained degrees	
Nostalgia proneness		15.90%	
Relatives and friends intensity		5.15%	
Emotional intensity		3.57%	
Trust	Emotional intensity-trust	9.86%	6.10%
	Relatives and friends intensity-trust		3.76%
Relationship commitment	Emotional intensity-commitment	62.88%	34.92%
	Trust -commitment		18.84%
	Relatives and friends intensity-trust		9.12%
Online donation willingness		79.92%	

(1) Insecurity, past experience, loneliness, and recovery from grief have a certain impact on individual nostalgia proneness. Influenced by the outbreak of COVID-19, these factors are the most representative, but there are other factors that are yet to be excavated.

As it can be seen in Table 5, the empirical results in this study show that the structural equation model has explained 15.9% of the variance of individual nostalgia proneness. It means that the exogenous variables (such as insecurity, past experience, loneliness, and recovery from grief) together explain 15.9% of the individual nostalgia proneness variance. The purpose of our study is not to explain all the influencing factors of individual nostalgia proneness. The variance explained that the degree also reached the middle according to Cohen (1992). Therefore, we think that the model is acceptable. From another perspective, it can be inferred that we also need to explore more influencing factors of individual nostalgia in future studies since the variance that explained the degree of individual nostalgia is not big enough.

(2) In donation scenarios, given nostalgia intensity includes two dimensions, relative and friend intensity and emotional intensity, which have a positive impact on nostalgia proneness. On the one hand, there may be other dimensions of nostalgia intensity; on the other hand, there may be other regulatory variables besides the influencing factors of nostalgia proneness.

As shown in Table 5, the results of this empirical study indicate that the structural equation model explains 5.15% of relative and friend intensity variance and 3.57% of emotional intensity variance. According to the standards of Cohen (1992), the variance explained that the degrees of nostalgic intensity exceed the smaller standard (2%), while there is a little gap (15%) from the medium. Therefore, this study is barely acceptable from the nostalgic intensity of this variable.

However, because the research on nostalgia intensity in the nostalgia study field is still at the exploratory stage, this article tends to separate nostalgia proneness from nostalgia intensity. It is considered an innovation itself to measure the nostalgia intensity with the intensity of relatives and friends and emotional intensity. In future research, first, there may also be other dimensions in the structure of nostalgia intensity besides relative and friend intensity and emotional intensity. Second, the two variables, relative and friend intensity and emotional intensity, are more complex, and they may also be influenced by many other psychological variables.

Based on the background of COVID-19, we found that, in addition to improving the performance of charity organizations, timely feedback, and effective communication, the donor’s individual psychological variables mentioned in this article are important factors in the source of trust of charitable organization donors (Sargeant et al., 2006). The more nostalgic the donors are, the stronger their trust on charities. We suggest that trust has a great influence on donation, and that trust will affect online donation willingness by affecting the relationship commitment of “donor–organization”. First of all, this is because COVID-19 and its corresponding avoidance measures make online donation the main force of donation channel, and the uncertainty of donor’s information is more ambiguous. The individual emotion plays an important role at this time. Nostalgia is the most prominent emotion in COVID-19.

As can been seen in Table 5, the empirical results of this study have shown that the structural equation model has explained 9.86% of the trust variance. The variance explained that the degree of emotional intensity to trust is 6.1% and that of relative and friend intensity is 3.76%. In other words, for the trust of donors on charitable organizations, stimulating the nostalgic feelings of donors is another way to improve the trust degree of the charitable organizations besides enhancing their own performance and effectively communicating. This study has demonstrated that the donors show more trust on charitable organization when their nostalgia proneness performs as missing relatives and friends under the stimulation of the charitable organizations and showing a strong sense of warmth after giving the donation. In order words, donors would reduce the sensitivity on trust variable in their donation decision-making behaviors.

(3) The change of donation channels highlights the role of relationship commitment. From the perspective of relationship marketing, relationship commitment is the most critical variable in determining consumer purchasing behavior. In the scene of the charitable donations, the relationship commitment of donors on charitable organizations has a remarkable impact on online donation willingness and donation behaviors of donors. The source of relationship commitment in donation context involves emotional intensity, followed by trust, and comes up with relative and friend intensity. Although trust has a great influence on relationship commitment and online donation willingness, the stimulation of nostalgic feelings (including other feelings that have not been researched) of the donors should be subject to the attention of managers of charitable organizations, especially in the COVID-19 era with nostalgia.

As shown in Table 5, the empirical results show that the structural equation model explains 62.88% of the relationship commitment variance, of which 34.92% comes from emotional intensity, 18.84% comes from trust, and 9.12% comes from friend and relative intensity, while 79.92% of the online donation willingness variance has been explained by the relationship commitment. According to the standards of Cohen (1992), the variance that explained the degree of relationship commitment and online donation willingness is more than 35%, which far outnumbers the standard (35%). Therefore, these two variables can be accepted in this study.

In the context of relationship marketing, the relationship between consumers and companies determines purchase intention and even brand loyalty. It means that the commitment of consumers to brand is considered an important channel promise such that enterprises launch brand management and enhance brand equity. In terms of this study, relationship commitment is a decisive factor in online donation willingness of donors. The relationship commitment level directly determines whether donors would like to donate money (goods) to charitable organizations and even to be willing to do long-term and ongoing donation. Therefore, the relationship commitment level directly determines the development of charitable organizations. Therefore, charitable organizations during the process of management should attach great importance to this variable.

Where is the source of relationship commitment? This study has demonstrated that nostalgia intensity has explained 34.04% of the variance of relationship commitment and that trust has explained 18.84% of the variance of commitment relationship. According to the data above, two conclusions can be inferred as follows:

First, trust has a great impact on relationship commitment. The level of trust directly affects the relationship commitment level of “donors to organization”. In the current donation environment in China, the public has shown the decreasing trust on charitable organizations because of their opaque information and corruption of their managers. Since the event of Guo Meimei, the number of donations that the Red Cross has received has witnessed a drastic reduction, which showed that the level of trust of donors on charitable organizations has a significant impact on the development of philanthropy in China.

Second, the nostalgic feelings of donors make greater contribution to relationship commitment. If charitable organizations feel like improving the level of donation, they should focus more on mental communication with donors and inspire their nostalgic feelings (including other feelings that have not been researched). In research on donation motivation, donation behaviors of donors are dominated by many psychological factors (such as nostalgia, pity, and guilt.), and studies on these emotional factors have an important influence on improving the level of charitable organizations as a whole.

### Managerial Implications

Charitable organizations can develop various strategies to provide a platform for making donors experience nostalgia, which will allow institutions and existing donors to establish stronger links and attract new donors, thereby benefiting the whole organization. Concretely speaking, charitable organizations, such as charity organizations of medical care and education, should take the nostalgic feelings of donors into consideration and effectively use them in marketing communication strategy development, market segmentation, target market selection, and positioning.

(1) Marketing managers of charitable organizations could design nostalgic communication demands, develop nostalgic platform, and evoke the nostalgic feelings of donors to increase online donation levels and willingness.

Marketing managers of charitable organizations must identify the important personal experiences of online donors and create a reasonable design in marketing communications (such as public service advertising), which develops a platform for online donors to allow them to re-experience the past and appeal to their personal nostalgic feelings. This kind of nostalgic communication strategy design is extremely effective in university alumni associations, hospitals, schools, poverty alleviation educational foundations, and any health-related charitable organizations.

Take the University Celebration Committee for example, in order to improve the online donation willingness and donation levels of schoolfellows, if the celebration preparatory groups mail the same paper and audio and video products to the schoolfellows that span decades or even centuries, it is impossible to produce a communication effect and make the school fellows produce nostalgic feelings or era resonance. For universities, they must segment schoolfellows according to the standard of admission age to raise the number of donations, since schoolfellows experienced different events during their school lives. Alumni associations should design different communication elements in various media (letters, email, audio and video products, photos, etc.), tell about dramatic events during their school age, give an account of their campus anecdotes, and record the characters and trivia of the faculty of donors and photos of canteens, dormitories, school buildings, libraries and campus, and other important landmark attractions. Although alumni associations cannot guarantee that each schoolfellow would receive a different media package (including the communication materials of different media), the alumni associations at least should be in accordance with the enrollment year and use 4 years as a unit so that the schoolfellows of different times could receive the unique materials related to their college lives and (cannot make it can be relaxed to 10 years). On the one hand, schoolfellows would feel valued; on the other hand, precisely because these materials (such as photos and audio and video products) are closely related to their college campus lives, they can effectively recall their college life and produce nostalgic feelings, which are accompanied by pleasantness and warmth, thereby enhancing the trust of schoolfellows, the relationship commitment degree, increasing online donation willingness, and even extending the loyalty on long-term donation.

(2) Managers of online charitable organizations should carry out effective market segmentation and positioning through nostalgic feelings.

Different people have different nostalgia proneness. Would nostalgic appeal have a negative impact on online donors who have low nostalgic feelings? Holbrook and Schindler (1991) suggested that consumers with high nostalgia proneness are more open and more easily accept nostalgic appeals (such as nostalgic advertising) rather than those with low nostalgic proneness. Holbrook and Schindler (2003) suggested that more progressive nostalgic appeals should be used in corporate marketing because the appeals could both evoke the traditional sense of the product or brand and display their modernity.

Therefore, if nostalgic appeal could stimulate nostalgic feelings of online donors, managers of charitable organizations must assess the nostalgia proneness of donors. This study has shown that online donors who are lonely and experiencing more significant past time, insecurity, and recovery from grief tend to easily become nostalgic. Managers of charitable organizations need to comprehensively assess these factors, which would enable to help them select the target market and identify real relevant online donors more easily by producing nostalgic feelings. However, in the management practice of charitable organizations, there is a difficult problem: how do charitable organizations obtain the personality of online donors, scenes of past life, and other factors? Gielens and Steenkamp (2007) provided an effective way to solve this problem. In the scenario used by new products, they require a certain brand community club of consumers to fill in the innovative scale and distinguish consumers into a noble new club and a low new club through the innovation scale. With companies designing different promotional packages, appeals of the new products would be served to the noble new groups. In the scenario of online donations, managers of charitable organizations enable to take advantage of a variety of scales (such as loneliness, insecurity, and past experience) in this study to measure the nostalgic feelings of donors, thereby distinguishing the high nostalgia proneness and low nostalgia proneness of online donors by target market selection, identifying target market selection, and finding out more relevant nostalgic appeal of donors. According to results of market segmentation and target market selection, charitable organizations could conduct more effective market position and set up a unique image bearing in the mind of online donors, thus to enhance their competitiveness in the competition.

(3) Managers of charitable organization should expand their impact on marketing and enhance online donation willingness and levels by nostalgia clubs (brand clubs). The greater the nostalgia intensity of online donors, the greater their relationship commitment to charitable organizations. Online donors with similar commitment gather in a virtual or real community in many ways, such as Wechat groups, forums, and so on, and could share past experiences with each other. Managers of charitable organizations need to build such community, organize recurrent activities or initiate discussions and communication, form a social organization, and inspire the nostalgic feelings of online donors, which would enhance the emotional contract and relationship commitment of the entire community to the charitable organizations. In the management practice of charitable organization, there are many small charitable organizations through the network platform, such as websites, forums, micro blogs, Wechat groups, and full discussions and communication, that stimulate nostalgic feelings of whole community, thereby improving the online donation willingness and donation levels of the entire organization.

## Limitations and Further Research

### Limitations

(1) The variance that explained the degree of nostalgic proneness and nostalgia intensity is limited. In the empirical model, exogenous variables (such as insecurity, past experiences, loneliness, and recovery from grief) together only explain 15.9% of the individual nostalgia proneness variance. Nostalgia proneness only explains 5.15% of the variance of friend and relative intensity and 3.57% of emotional intensity. According to the standard of Cohen (1992), the explained degree is small or just reached medium.

(2) It is possible that there exist other variables between nostalgia intensity and relationship commitment. Charitable organizations may take some actions, including writing response cards and letters of thanks to increase the emotional contract and relationship commitment of donors on charitable organizations, which requires further research to explore.

(3) It is possible that there exists other structure dimensions in nostalgia intensity. It is difficult to distinguish emotional intensity from relative and friend intensity in nostalgia intensity. However, emotional intensity is only related to positive emotions, so future studies may also need to focus on other dimensions of nostalgia intensity as well as negative nostalgic feelings.

(4) Limitations of the data and scales. Although some of the scales meet the requirements, their reliability is not high, such as insecurity in nostalgia proneness, relative and friend intensity, and emotional intensity in nostalgia intensity, which indicates that there is room for improvement in the scales themselves. The process of the small sample investigation and formal investigation and the geographical scope of sample survey is relatively narrow, which may affect the universality of the data. The number of valid questionnaires is also limited. Although they meet the statistical requirements, the proportion of valid questionnaires in the course of the investigation is not high, which has some impact on this study.

### Further Research

(1) Further exploration on influencing factors of nostalgic proneness is needed. Since the exogenous variables only explain 15.9% of the variance of individual nostalgia proneness, it can be inferred that more influencing factors of individual nostalgia should be explored in future studies. Moreover, further exploration of collective (substitution) nostalgia should be discussed on donation behavior.

(2) Further exploration on dimensionalities and influencing factors of nostalgia intensity is needed. The variance that explained the degree of nostalgia intensity is lower. For future research, first, the structure of nostalgia intensity may also show other dimensions that need to be explored besides the intensity of relatives and friends and emotional intensity. Second, because the two variables, relative and friend intensity and emotional intensity, are more complex, they may also be influenced by many other psychological variables.

(3) Improvements of the empirical survey data on quantity and quality is required. First of all, due to insecurity in nostalgia proneness and relative and friend intensity and the emotional intensity in nostalgia intensity, there are problems on the scales themselves and that they need further improvements. Second, the number of the survey questionnaires and the proportion of valid questionnaires are limited. Under the conditions of human, material, and financial resources in the future, improvement of survey data could be enhanced from quantity and quality. In the empirical study, cross-sectional data are used, and there would be great differences between different charitable organizations, so it is not appropriate to study and infer that nostalgic feelings have a significant effect on all donation behaviors. In future studies, we can demonstrate the relationship between nostalgic feelings and donation behavior of different organizations (such as alumni associations and various disease foundations).

(4) There exist many other influencing factors that need to be developed about online donation willingness. For example, what roles do response cards and letters of thanks that donors receive play in the model of donation decision-making? Does receiving letters of thanks have a significant impact on future donations? Is there any difference in the attitude on letters of thanks between donors with low frequency and high frequency? In addition, is it possible that donation behaviors stem from religious beliefs or donation habits? Would they last? Would the habit be interrupted by indifferent attitudes charity organizations hold? Would the relationship commitment of donors be improved if charitable organizations promise them in advance? Further research is needed to demonstrate the abovementioned issues.

## Data Availability Statement

The raw data supporting the conclusions of this article will be made available by the authors, without undue reservation.

## Author Contributions

YZ was responsible for determining the theme and completed most of the writing. WT was responsible for issuing and collecting the data and completed data analysis. Both authors contributed to the article and approved the submitted version.

## Conflict of Interest

The authors declare that the research was conducted in the absence of any commercial or financial relationships that could be construed as a potential conflict of interest.

## Publisher’s Note

All claims expressed in this article are solely those of the authors and do not necessarily represent those of their affiliated organizations, or those of the publisher, the editors and the reviewers. Any product that may be evaluated in this article, or claim that may be made by its manufacturer, is not guaranteed or endorsed by the publisher.
